# Dietary Patterns Are Not Consistently Associated with Variability in Blood Lead Concentrations in Pregnant British Women

**DOI:** 10.1093/jn/nxz023

**Published:** 2019-04-10

**Authors:** Caroline M Taylor, Rita Doerner, Kate Northstone, Katarzyna Kordas

**Affiliations:** 1 Centre for Academic Child Health, Bristol Medical School, University of Bristol, UK; 2 Bristol Medical School, University of Bristol, UK; 3 Epidemiology and Environmental Health, School of Public Health and Health Professions, University at Buffalo, Buffalo, NY

**Keywords:** pregnancy, dietary patterns, lead, prenatal, blood lead, ALSPAC

## Abstract

**Background:**

During pregnancy lead crosses the placenta freely and can have adverse effects on the fetus, with the potential for lifelong impact on the child. Identification of dietary patterns and food groups in relation to measures of lead status could provide a more useful alternative to nutrient-specific advice to minimize fetal lead exposure.

**Objectives:**

The aim of this study was to evaluate whether dietary patterns and food groups are associated with blood lead concentration (B-Pb) in pregnancy.

**Design:**

Whole blood samples were collected at a median of 11 wk gestation (IQR 9–13 wk) from women enrolled in the Avon Longitudinal Study of Parents and Children birth cohort study, and analyzed for lead. Dietary pattern scores were derived from principal components analysis of a food-frequency questionnaire (32 wk gestation). Associations of dietary pattern scores (quartiles), and of food groups (frequency of consumption), with the likelihood of B-Pb ≥5 µg/dL identified with adjusted logistic regression (*n* = 2167 complete cases).

**Results:**

There was a negative association between the “confectionery” dietary pattern and the likelihood of B-Pb ≥5 µg/dL (OR: 0.62; 95% CI: 0.41, 0.94) in an adjusted model. There were no associations with other dietary patterns. There was a positive association between the food group “all leafy green and green vegetables” and the likelihood of B-Pb ≥5 µg/dL (OR 1.45; 95% CI: 1.04, 2.01). Conversely, the food group “cakes and biscuits” was negatively associated (OR 0.63; 95% CI: 0.43, 0.93). After multiple imputation, there was a positive association of the “healthy” diet pattern and no association of the “confectionery” pattern.

**Conclusions:**

We found limited evidence of an association between women's typical diet and B-Pb during pregnancy. Our findings do not indicate need to revise dietary guidance for pregnant women, who are advised to adopt a healthy diet in pregnancy, with a variety of foods consumed in moderation.

## Introduction

Lead is a widespread and persistent environmental contaminant derived from natural sources and from anthropogenic activities such as mining, smelting, and manufacturing. It is a neurotoxin with adverse effects on many aspects of human health, including reproductive functions, birth outcomes, and child development (1–4). Lead abatement measures adopted in most countries in recent years, including the removal of lead from paint and petrol, have resulted in declines in lead concentrations in children and adults (5, 6). However, there is increasing awareness that adverse effects occur at all levels of lead exposure, with no lower limit (7–9).

Exposure to lead can occur through food, water, dust, soil, and air. Currently, the main route of exposure for adults in Europe is diet, although dust and soil remain important sources for children (7). Cereals and vegetables, particularly leafy vegetables, are the greatest contributors to dietary lead intake in Europe (7, 10). Diet can influence the overall lead burden in 2 main ways: *1*) through the lead content of individual food items; and *2*) by the modification of lead absorption and excretion by individual nutrients and by the physiochemical properties of the food (11). For example, calcium and iron intakes appear to be inversely associated with blood lead concentrations (B-Pb) in pregnancy (12, 13), whereas vitamin D and phosphorus had positive associations with bone lead in middle-aged and elderly men (14). Indeed, advice aimed at reducing children's B-Pb in the United States focuses on the provision of foods rich in iron, vitamin C, and calcium (15, 16). It is difficult to estimate lead exposure from diet in a population due to lack of extensive food composition data and variations in the lead content of foods due to growing and processing conditions. Analysis of dietary patterns and food groups in relation to lead status could provide an alternative to nutrient-specific advice to enable a more readily interpretable approach to evaluating diet in regard to lead exposure.

Dietary patterns enable the study of the diet as a whole, thus allowing the inclusion of many foods in combination. Principal components analysis (PCA), in which underlying dimensions in the data are identified based on inter-item correlations, is the most frequently used method for examining dietary patterns (17). Dietary patterns identified via PCA were predictive of bone and B-Pb in elderly men in the United States and Sweden, and in Korean adolescents and adults (18–20). To our knowledge, dietary pattern analysis has not been undertaken for pregnant women in association with biomarkers of lead exposure: this is important because the fetus is vulnerable to the effects of lead due to the rapid development of the nervous system (21, 22). During pregnancy, lead crosses the placenta freely (23, 24) and can adversely affect a range of birth outcomes, with the potential for lifelong impact on the growth and development of the child (21).

The UK Avon Longitudinal Study of Parents and Children (ALSPAC) is a longitudinal birth cohort study in which lead exposure was measured as the mothers’ B-Pb during pregnancy and dietary data were collected via an FFQ. Five dietary patterns have been determined previously for these women through the use of PCA (25, 26). The aim of the present study was to evaluate whether these dietary patterns are associated with B-Pb in pregnancy. An additional aim was to evaluate the role of specific food groups in predicting B-Pb concentrations.

## Methods

The sample for this analysis was derived from ALSPAC, a UK-based birth cohort set up to investigate environmental and genetic influences on health and disease. ALSPAC recruited 14,541 pregnant women residing in Avon, UK with expected dates of delivery between April 1, 1991 and December 31, 1992. The cohort profile of the mothers in ALSPAC is described in detail elsewhere (27). The study website contains details of all the data that are available through a fully searchable data dictionary (28). Ethics approval for the study was obtained from the ALSPAC Ethics and Law Committee and the Local Research Ethics Committees.

The primary source of data collection was self-completed questionnaires sent to the mother. Blood samples were collected by midwives during pregnancy. Of the 14,541 women enrolled in ALSPAC, 4484 provided a blood sample for analysis at a median of 11 wk gestation. Of these, 3748 provided dietary data at 32 wk gestation.

## Exposures

The nutritional exposures were collected via an FFQ sent to the ALSPAC mothers at 32 wk gestation (29). The FFQ was based on a questionnaire used previously in a neighboring area and on weighted intake data collection from nonpregnant women in the local area. The questionnaire asked about the frequency of consumption of 43 different foods and drinks. Possible answers were the following: *1*) never or rarely; *2*) once in 2 wk; *3*) 1–3 times/wk; *4*) 4–7 times/wk; or *5*) >1/d. This FFQ has been shown to produce mean nutrient intakes (30) similar to those obtained for women in the UK National Diet and Nutrition Survey for adults (31). It was not specifically validated, but data on fish consumption, for example, were related to blood concentrations of mercury and long-chain fatty acids (32, 33).

### Food groups

We combined individual foods into 10 food groups: *1*) meats; *2*) fish; *3*) pulses; *4*) nuts; *5*) soybean products; *6*) root vegetables; *7*) leafy greens and green vegetables; *8*) breads and cereals; *9*) cakes and biscuits; *10*) pastas and rice. The combinations of foods are described in **Supplemental Table 1**. According to the originally reported frequency of consumption per type of food, mothers were allocated to frequency categories for the new combined food groups. As consumption often varied by food type, mothers were allocated to the subcategory representing the most frequent consumption. For example, within “meats” where a mother reported the consumption of poultry to be “1–3 times/wk” and red meat to be “4–7 times/wk”, she would be assigned to “4–7 times/wk” for the combined “meats” group.

### Dietary patterns

For a separate analysis, we used dietary patterns, which had been derived in this population from 43 standardized food items, as described in detail elsewhere (25). Briefly, the number of components best representing the data was chosen on the basis of a scree plot and the interpretability of the patterns. A component score was created for each woman for each of the components identified. The scores are a weighted linear combination of the food items, such that the weights determine how important each food item is for that score. Each score has a mean of 0 and a higher score indicates closer adherence to that dietary pattern. Five components were obtained (25): “health conscious” (high factor loadings, >0.3, for salad, fruit, rice, pasta, oat- and bran-based breakfast cereals, fish, pulses, fruit juices and nonwhite bread); “traditional” (high consumption of all types of vegetables and red meat and poultry); “processed” (high intakes of high-fat processed foods, such as meat pies, sausages and burgers, fried foods, pizza, chips, and baked beans); “confectionery” (high intakes of foods with high sugar content, such as chocolate, sweets, biscuits, cakes, and other puddings); and “vegetarian” (high loadings for meat substitutes, pulses, nuts, and herbal teas, and high negative loadings for red meat and poultry).

### Outcome

#### Collection, storage, and analysis of blood samples

Whole blood samples were collected by midwives as early as possible in pregnancy in a subsample of women who gave consent. One district within the sample area was unable to participate because their sampling system was not compatible with ALSPAC requirements. Details of the sample collection and analyses are shown in the **Supplemental Methods**. The laboratory assays were completed on 4285 samples. B-Pb concentration values were dichotomized (<5 and ≥5 µg/dL), based on previously reported US cutoffs indicating “reference” concentrations of B-Pb (34, 35).

## Potential confounders

Potential confounding factors were defined a priori from the literature. Details of the confounders are given in the **Supplemental Matter**.

## Statistical analysis

We initially examined potential response bias in the sample by comparing mothers who completed the FFQ and had available data on B-Pb with those who did not. The associations between B-Pb and background characteristics were examined.

Univariable and multivariable logistic regression modeling was used to examine the likelihood of having B-Pb ≥5 µg/dL in cases with complete exposure, outcome, and confounder data only. Two sets of models were produced: one set included the dietary pattern scores as exposures and a second set the food groups. Within each set, model 1 was unadjusted and model 2 was adjusted for indicators of socioeconomic status, BMI, energy intake, smoking status, alcohol consumption, and hemoglobin concentrations. For the set of models with dietary pattern scores, an additional model (model 3) included mutual adjustment for dietary pattern scores. The overall contribution of the exposure to every model was assessed with likelihood ratio tests at each stage of the process. Results are reported as unadjusted and adjusted ORs with 95% CIs and *P* values.

Univariable and multivariable regression modeling was also used to examine the linear association of continuous log B-Pb concentration with the dietary outcomes for complete cases.

**Figure 1:**
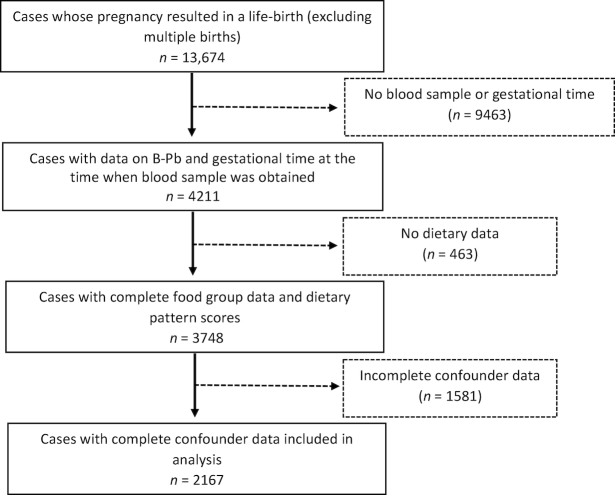
Study flow chart.

Of the 13,674 cases with a live birth, 9463 had no blood sample available for analysis, or had missing data for gestation time, or both. A further 463 had no dietary data, and a further 1581 participants had missing data on covariates (**Figure 1**). Thus, further analyses were conducted to examine the influence of missing data on the findings. Multiple imputation by chained equation was used to impute missing data with the use of the ice command in STATA (36). We generated 25 data sets and undertook 10 switching procedures. The following variables were used to impute: all outcomes, main predictors, and other variables included in the adjusted analyses plus those included in the Family Adversity Index, and the (binary) Strengths and Difficulties Questionnaire (high/low) indicators, and indicators of family adversity: early parenthood, housing [adequacy, basic living facilities (e.g., hot water), defects], financial difficulties, partner (present/absent), relationship with partner (affection/cruelty/support), family size, major family problems (child in care, not with natural mother or on at risk register), maternal depression/anxiety, substance abuse, crime (trouble with police or convictions). The analyses of associations between dietary patterns and food groups and B-Pb were then repeated with the imputed data Figure 1.

Statistical analysis was conducted with STATA version 14.2 (StataCorp).

## Results

Of the mothers whose pregnancy resulted in a live birth (*n* = 13,674), 4211 had data on B-Pb and gestational age at the time that the sample was taken. On further exclusion of cases without dietary data or confounders, 2167 cases were left for complete case analysis. The study flow chart is shown in Figure 1. The mean ± SD B-Pb was 3.64 ± 1.46 µg/dL (median: 3.37; IQR: 1.68 µg/dL). The mean ± SD gestational age at which blood samples were drawn was 11.0 ± 4.0 wk. The number of participants included or excluded in the analyses is shown in **Table 1**. In brief, the participants included in the study were more likely to be older and not to smoke, have higher educational attainment and social class, and have a higher Townsend score (an indicator of deprivation). The demographic characteristics according to B-Pb category (<5 or ≥5 µg/dL) are shown in **Table 2**. Participants with B-Pb ≥5 µg/dL were more likely to be older, have higher education and a higher Townsend score, smoke, and be vegetarian compared with participants with B-Pb <5 µg/dL.

**TABLE 1:** Characteristics of included/excluded ALSPAC participants^1^

	*n* (%)			
Participant characteristic	Excluded	Included	OR (95% CI)	*P* value
	11,507/13,674 (84.2)	2167/13,674 (15.9)	—	
Maternal age at pregnancy, y				<0.001
Total *n*	11,507	2167	—	
≤19	600 (5.2)	53 (2.5)	1.00	
20–<25	2341 (20.3)	313 (14.4)	1.51 (1.11, 2.05)	
25–<30	4437 (38.6)	850 (39.2)	2.17 (1.62, 2.90)	
30–<35	3016 (26.2)	709 (32.7)	1.66 (1.99, 3.57)	
≥35	1113 (9.7)	242 (11.2)	1.46 (1.80, 3.37)	
Maternal education				<0.001
Total *n*	9969	2167	—	
None/CSE	3105 (31.2)	540 (24.9)	1.00	
Vocational/O-level	5672 (56.9)	1262 (58.2)	1.28 (1.15, 1.43)	
A-level and above	1192 (12.0)	365 (16.8)	1.76 (1.52, 2.04)	
Maternal social class^2^				0.077
Total *n*	7961	1871	—	
I/II	2937 (36.9)	733 (39.2)	1.00	
III nonmanual/III manual	4042 (50.8)	935 (50.0)	0.92 (0.83, 1.03)	
IV/V	982 (12.3)	203 (10.9)	0.83 (0.70, 0.98)	
Paternal social class				0.056
Total *n*	8700	1996	—	
I/II	3851 (44.3)	963 (483)	1.00	
III nonmanual/III manual	3734 (42.9)	794 (39.8)	0.85 (0.77, 0.94)	
IV/V	1115 (12.8)	239 (12.0)	0.86 (0.73, 1.00)	
Maternal Townsend score^3^				<0.001
Total *n*	7214	2167	—	
1	2347 (32.5)	658 (30.4)	1.00	
2	1399 (19.4)	340 (15.7)	0.87 (0.75, 1.00)	
3	1908 (26.5)	577 (26.6)	1.08 (0.95, 1.22)	
4	1560 (21.6)	592 (27.3)	1.36 (1.19, 1.54)	
Maternal smoking status in first trimester				0.001
Total *n*	10,712	2167	—	
No	7975 (74.5)	1685 (77.8)	1.00	
Yes	2737 (25.6)	482 (22.2)	0.83 (0.75, 93)	
Maternal dietary scores			—	
Total *n*	9670	2167	—	
Healthy	−0.024 ± 1.00	0.100 ± 0.98	—	0.000
Traditional	−0.006 ± 1.00	0.009 ± 0.97	—	0.531
Processed	0.011 ± 1.01	−0.055 ± 0.94	—	0.006
Confectionery	0.008 ± 1.01	−0.023 ± 0.96	—	0.201
Vegetarian	−0.002 ± 0.99	0.009 ± 1.03	—	0.643

1 ALSPAC, Avon Longitudinal Study of Parents and Children; CSE, Certificate of Secondary Education.

2 Social class: I, higher managerial, administrative, or professional; II, intermediate managerial, administrative or professional; III, nonmanual, supervisory or clerical and junior management, administrative or professional; III, manual, skilled manual workers; IV, semiskilled and unskilled manual workers; V, casual or lowest-grade workers.

3 The Townsend score is a measure of deprivation: 1 = least deprived; 4 = most deprived (37).

**TABLE 2:** B-Pb concentrations of ALSPAC participants by indicators of socioeconomic status and lifestyle^1^

	Observations included in sample, *n* (%)	*n* (%)			
Variable		B-Pb <5 µg/dL	B-Pb ≥5 µg/dL	OR (95% CI)	*P* value
Total *n*^2^	2167	1861	306	—	
Maternal age at pregnancy, y					<0.001
≤19	53 (2.5)	42 (2.3)	11 (3.6)	1.00	
20–<25	313 (14.4)	284 (15.3)	29 (9.5)	0.66 (0.38, 1.15)	
25–<30	850 (39.2)	739 (39.7)	111 (36.3)	0.89 (0.53, 1.48)	
30–<35	709 (32.7)	608 (32.7)	101 (33.0)	1.03 (0.61, 1.72)	
≥35	242 (11.8)	188 (10.1)	54 (17.7)	1.62 (0.94, 2.78)	
Maternal education (at pregnancy)					<0.001
None/CSE	478 (24.9)	478 (25.7)	62 (20.3)	1.00	
Vocational/O-level	1098 (58.2)	1098 (59.0)	164 (53.6)	1.15 (0.84, 1.57)	
A-level and above	285 (16.8)	285 (15.3)	80 (26.1)	2.16 (1.51, 3.11)	
Townsend score (at pregnancy)^3^					<0.001
1	658 (30.4)	606 (32.6)	52 (17.0)	1.00	
2	340 (15.7)	296 (15.9)	44 (14.4)	1.73 (1.13, 2.65)	
3	577 (26.6)	549 (24.7)	118 (38.5)	3.00 (2.12, 4.24)	
4	592 (27.3)	500 (26.9)	92 (30.1)	2.14 (1.50, 3.07)	
Smoking status (first trimester)					<0.001
No	1685 (77.8)	1479 (79.5)	206 (67.3)	1.00	
Yes	482 (22.2)	382 (20.5)	100 (32.7)	1.88 (1.44, 2.45)	
Alcohol consumption (first trimester)					0.057
No	958 (44.2)	838 (45.0)	120 (39.2)	1.00	
Yes	1209 (55.8)	1023 (55.0)	186 (60.8)	1.27 (0.99, 1.63)	
BMI (prepregnancy)^4^					0.813
Normal/underweight	1709 (78.9)	1465 (78.8)	244 (79.7)	1.00	
Overweight	343 (15.8)	295 (15.9)	48 (15.7)	0.98 (0.70, 1.36)	
Obese	115 (5.3)	101 (5.4)	14 (4.6)	0.83 (0.47, 1.48)	
Vegetarian diet^2^					0.004
Never	1810 (85.3)	1572 (86.4)	238 (78.8)	1.00	
In the past	180 (8.5)	144 (7.9)	36 (11.9)	1.65 (1.12, 2.44)	
Present	131 (6.2)	103 (5.7)	28 (9.3)	1.80 (1.57, 2.79)	

1 ALSPAC, Avon Longitudinal Study of Parents and Children; B-Pb, blood lead concentration; CSE, Certificate of Secondary Education.

2 Total *n* for vegetarian diet = 2121 observations; B-Pb **<**5 µg/dL, *n* **= **1819; B-Pb **≥**5 µg/dL, *n* = 302.

3 The Townsend score is a measure of deprivation: 1 = least deprived; 4 = most deprived (37).

4 BMI: normal/underweight ≤24.9 kg/m^2^; overweight 25.0–29.9 kg/m^2^; obese ≥30.0 kg/m^2^.

The “confectionery” pattern was negatively associated with the likelihood of B-Pb ≥5 µg/dL in the adjusted model 2 (**Table 3**) (quartiles 3 and 4 compared with quartile 1). None of the other dietary patterns had any association with B-Pb. The likelihood ratio tests to compare the goodness of fit of the models showed that, in general, there was no advantage in mutual adjustment for each dietary pattern, but the association for the “confectionery” pattern was considerably weakened.

**TABLE 3:** Categoric B-Pb of cases with B-Pb measurements by dietary patterns (complete case analysis; *n* = 2167)^1^

		Median B-Pb (µg/dL)	*n* (%)		OR (95% CI)		
Pattern	Quartile		B-Pb <5 µg/dL	B-Pb ≥5 µg/dL	Unadjusted model 1	Adjusted model 2	Adjusted model 3
Total *n*		—	1861	306	—	—	—
Healthy	1	3.46	398 (21.4)	60 (20.0)	1.00	1.00	1.00
	2	3.31	450 (24.2)	70 (22.9)	1.03 (0.71, 1.49)	1.16 (0.78, 1.73)	1.20 (0.81, 1.81)
	3	3.29	516 (27.7)	78 (26.0)	1.00 (0.70, 1.44)	1.16 (0.76, 1.75)	1.22 (0.80, 1.88)
	4	3.40	497 (26.7)	98 (32.0)	1.31 (0.92, 1.85)	1.44 (0.91, 2.26)	1.53 (0.94, 2.50)
	—	—	—	—	*P*-trend = 0.134	*P*-trend = 0.139	*P*-trend = 0.110
Traditional	1	3.29	465 (25.0)	68 (22.2)	1.00	1.00	1.00
	2	3.31	440 (23.6)	70 (22.9)	1.09 (0.76, 1.56)	1.12 (0.77, 1.65)	1.14 (0.79, 1.68)
	3	3.52	479 (25.7)	86 (28.1)	1.23 (0.87, 1.73)	1.27 (0.89, 1.83)	1.32 (0.92, 1.90)
	4	3.33	477 (25.6)	82 (26.8)	1.18 (0.83, 1.66)	1.27 (0.88, 1.83)	1.29 (0.89, 1.89)
	—	—	—	—	*P*-trend = 0.281	*P*-trend = 0.159	*P*-trend = 0.137
Processed	1	3.38	485 (26.1)	81 (26.5)	1.00	1.00	1.00
	2	3.47	484 (26.0)	85 (27.8)	1.05 (0.76, 1.46)	1.14 (0.80, 1.61)	1.14 (0.80, 1.63)
	3	3.33	448 (24.1)	67 (21.9)	0.90 (0.63, 1.27)	1.02 (0.70, 1.50)	1.04 (0.70, 1.54)
	4	3.33	444 (23.9)	73 (23.9)	0.98 (0.70, 1.39)	1.35 (0.89, 2.03)	1.36 (0.86, 2.15)
	—	—	—	—	*P*-trend = 0.707	*P*-trend = 0.267	*P*-trend = 0.313
Confectionery	1	3.48	443 (23.8)	103 (33.7)	1.00	1.00	1.00
	2	3.37	479 (25.7)	83 (27.1)	0.75 (0.54, 1.02)	0.80 (0.57, 1.12)	0.83 (0.59, 1.17)
	3	3.33	477 (25.6)	65 (21.2)	0.59 (0.42, 0.82)	0.66 (0.46, 0.95)	0.72 (0.49, 1.06)
	4	3.30	462 (24.8)	55 (18.0)	0.51 (0.36, 0.73)	0.62 (0.41, 0.94)	0.73 (0.46, 1.16)
	—	—	—	—	*P*-trend < 0.001	*P*-trend = 0.011	*P*-trend = 0.108
Vegetarian	1	3.27	507 (27.2)	72 (23.5)	1.00	1.00	1.00
	2	3.37	476 (25.6)	67(21.9)	0.99 (0.69, 1.41)	0.87 (0.6, 1.27)	0.86 (0.59, 1.27)
	3	3.27	436 (23.4)	75 (24.5)	1.21 (0.86, 1.72)	1.09 (0.75, 1.58)	1.14 (0.78, 1.66)
	4	3.57	442 (23.8)	92 (30.1)	1.47 (1.05, 2.05)	1.14 (0.80, 1.64)	1.17 (0.82, 1.68)
	—	—	—	—	*P*-trend *=* 0.013	*P*-trend = 0.265	*P*-trend = 0.194

1 B-Pb, blood lead concentration. Model 2 adjusted for maternal age, maternal education, Townsend score + BMI and energy intake + alcohol consumption and smoking status during first trimester + hemoglobin levels.

Model 3 adjusted for maternal age, maternal education, Townsend score + BMI and energy intake + alcohol consumption and smoking status during first trimester + hemoglobin concentrations + dietary pattern scores.

With regard to food groups (**Table 4**), 1 group was predictive of B-Pb ≥5 µg/dL in the adjusted model 2: “all leafy green and green vegetables” consumption for ≥4 times/wk compared with ≤1 to 3 times/wk. Conversely, 1 group was negatively associated with B-Pb ≥5 µg/dL in the adjusted model: “all cakes and biscuits.” None of the other food groups had any association with B-Pb in the adjusted models.

**TABLE 4:** Categoric B-Pb of cases with valid B-Pb measurements by food group (complete case analysis, *n* = 2167)^1^

	*n* (%)		OR (95% CI)	
	B-Pb <5 µg/dL	B-Pb ≥5 µg/dL	Unadjusted model 1	Adjusted model 2
Total *n*^2^	1861	306	—	—
All meats combined				
≤Once in 2 wk	247 (13.3)	52 (16.99)	1.00	1.00
≤3 times/wk	1181 (63.5)	182 (59.48)	0.73 (0.52, 1.03)	0.88 (0.61, 1.26)
≥4 times/wk for ≥1 group	433 (23.3)	72 (23.53)	0.79 (0.54, 1.17)	1.06 (0.69, 1.63)
	—	—	*P*-trend = 0.351	*P*-trend = 0.695
All fish				
≤Once in 2 wk	926 (49.7)	154 (50.33)	1.00	1.00
≥1–3 times/wk	870 (46.8)	135 (44.12)	0.93 (0.73, 1.19)	0.99 (0.77, 1.30)
≥4–7 times/wk	65 (3.5)	17 (5.56)	1.57 (0.90, 2.75)	1.66 (0.91, 3.03)
	—	—	*P*-trend = 0.671	*P*-trend = 0.388
Milk, glasses/d^3^				
None/rarely	700/1810 (42.5)	148/291 (50.86)	1.00	1.00
1–2 glasses/d	879/1810 (48.6)	126/291 (43.3)	0.75 (0.58, 0.96)	0.81 (0.62, 1.06)
≥3 glasses/d	161/1810 (8.9)	17/291 (5.84)	0.19 (0.32, 0.93)	0.64 (0.64, 1.11)
	—	—	*P*-trend = 0.004	*P*-trend = 0.046
Calcium intake (quartiles)				
1	427 (22.9)	89 (29.08)	1.00	1.00
2	483 (26.0)	88 (28.76)	0.87 (0.63, 1.20)	0.91 (0.64, 1.30)
3	482 (25.9)	66 (21.57)	0.66 (0.47, 0.93)	0.64 (0.42, 0.97)
4	469 (25.2)	63 (20.59)	0.64 (0.45 0.91)	0.73 (0.44, 1.21)
	—	—	*P*-trend = 0.004	*P*-trend = 0.089
All pulses combined				
≤Once in 2 wk	228 (12.3)	44 (14.38)	1.00	1.00
≤3 times/wk	1475 (79.3)	233 (76.14)	0.82 (0.58, 1.16)	0.84 (0.58, 1.22)
≥4 times/wk for ≥1 group	158 (8.5)	29 (9.48)	0.95 (0.57, 1.59)	0.95 (0.55, 1.64)
	—	—	*P*-trend = 0.687	*P*-trend = 0.736
All nuts combined				
Never/rarely	1210 (65.0)	186 (60.78)	1.00	1.00
≤Once in 2 wk	471 (25.3)	84 (27.45)	1.16 (0.881.53)	1.04 (0.77, 1.41)
≥1–3 times/wk	180 (9.7)	36 (11.76)	1.30 (0.88 1.92)	1.20 (0.78, 1.82)
	—	—	*P*-trend = 0.126	*P*-trend = 0.435
Soybean products				
Never or rarely	1697 (91.2)	267 (97.25)	1.00	1.00
≤Once in 2 wk	164 (8.8)	39 (12.75)	1.51 (1.04, 2.19)	1.35 (0.91, 2.00)
	—	—	*P*-trend = 0.030	*P*-trend = 0.138
Root vegetables				
Never or rarely	79 (4.3)	12 (3.92)	1.00	1.00
≤1–3 times/wk per food	1103 (59.3)	183 (59.8)	1.09 (0.58, 2.04)	1.09 (0.56, 2.09)
≥4–7 times/wk	679 (36.5)	111 (36.27)	1.08 (0.57, 2.04)	1.25 (0.63, 2.47)
	—	—	*P*-trend = 0.974	*P*-trend = 0.289
All leafy green and green vegetables				
≤1–3 times/wk	449 (24.1)	56 (18.3)	1.00	1.00
≥4 times/wk	1412 (75.9)	250 (81.7)	1.42 (1.04, 1.93)	1.45 (1.04, 2.01)
	—	—	*P*-trend = 0.026	*P*-trend = 0.028
Combined breads and cereals				
≤Once a week	213 (11.5)	50 (16.34)	1.00	1.00
≤1–3 times/wk per food	523 (28.1)	79 (25.82)	0.64 (0.44, 0.95)	0.71 (0.47, 1.07)
≥4–7 times/wk	1125 (60.5)	177 (57.84)	0.67 (0.47, 0.95)	0.85 (0.58, 1.24)
	—	—	*P*-trend = 0.083	*P*-trend = 0.776
All cakes and biscuits				
≤Once a week	310 (16.7)	78 (25.49)	1.00	1.00
≤1–3 times/wk per food	932 (49.6)	146 (47.71)	0.63 (0.46, 0.85)	0.68 (0.49, 0.94)
≥4–7 times/wk	628 (33.8)	82 (26.8)	0.52 (0.37, 0.73)	0.63 (0.43, 0.93)
	—	—	*P*-trend < 0.001	*P*-trend = 0.027
All pies and pastries				
Never or rarely	368 (19.8)	72 (23.53)	1.00	1.00
≤Once in 2 wk	1025 (55.1)	172 (56.21)	0.86 (0.66, 1.16)	0.97 (0.71, 1.34)
≥1–3 times/wk	468 (25.2)	62 (20.26)	0.68 (0.47, 0.98)	0.85 (0.57, 1.27)
	—	—	*P*-trend = 0.036	*P*-trend = 0.441
All pasta and rice				
Never or rarely	188 (10.1)	26 (8.5)	1.00	1.00
≤Once in 2 wk	577 (31.0)	76 (24.84)	0.95 (0.59, 1.53)	1.00 (0.61, 1.64)
≥1–3 times/wk	1096 (58.9)	204 (66.67)	1.34 (0.87, 2.08)	1.38 (0.86, 2.22)
	—	—	*P*-trend = 0.023	*P*-trend = 0.050

1 B-Pb, blood lead concentration. Model 2 adjusted for maternal age, maternal education, Townsend score + BMI and energy intake + alcohol consumption and smoking status during first trimester + hemoglobin concentrations.

2 Total *n* for vegetarian diet = 2121 observations; B-Pb **<**5 µg/dL, *n* **= **1819; B-Pb **≥**5 µg/dL, *n* = 302.

3 A standard glass of milk is 200 mL.

In the further analyses on the dataset with multiple imputation, the results were broadly similar (**Supplemental Tables 3** and **4**). However, for dietary patterns there was an additional association of the “healthy” pattern with B-Pb ≥5 µg/dL (Q4 versus Q1; OR: 1.58; 95% CI: 1.27, 1.97). With the additional mutual adjustment for dietary pattern scores (model 3), these associations remained. For food groups, after multiple imputation the negative association of “all cakes and biscuits” with B-Pb was not retained, nor was there any association with “all leafy green and green vegetables.” There was an additional negative association of milk consumption with B-Pb [none/rarely compared with >3 glasses (standard glass = 200 mL) per day; OR: 0.77; 95% CI: 0.61, 0.98].

With B-Pb as a continuous variable, there were no associations with any of the dietary patterns in the adjusted model. As in the categoric model, there was a negative association with “all cakes and biscuits,” and with “milk” (**Supplemental Tables 5** and **6**).

## Discussion

In this study of pregnant women with relatively low B-Pb, there was a weakly negative association between the “confectionery” dietary pattern and the likelihood of having B-Pb ≥5 µg/dL. There were no associations for the other dietary patterns. There was a strong positive association between the food group “all leafy green and green vegetables” and the odds of having a B-Pb ≥5 µg/dL. Conversely, “cakes and biscuits” had a negative association with B-Pb ≥5 µg/dL.

To our knowledge, there have only been 3 previous studies on dietary patterns in relation to biomarkers of lead exposure in adults. None of these were in pregnancy, and were based in countries with culturally differing dietary habits. In middle-aged and elderly men in the United States a “Western” diet pattern (characterized by high intakes of processed meat, red meat, refined grains, high-fat dairy products, French fries, butter, and eggs) was positively associated with B-Pb and bone lead. A “prudent” dietary pattern (characterized by higher intakes of fruit, legumes, vegetables, whole grains, poultry, and seafood) was not associated with bone or blood lead (20). In older Swedish men and women, a “low-carbohydrate/high-protein” pattern (characterized by high intakes of dairy products, meat and meat products, and fish) was positively associated with B-Pb, and a “WHO-recommended” pattern [characterized by high fruit and vegetable, low sugar and salt, and low fat, particularly saturated fat, intake (38)] was negatively related to B-Pb (18). In Korean adults, a “balanced diet” pattern (characterized by high intakes of vegetables, fish, meat, and milk) was negatively associated with B-Pb, whereas an “alcohol and noodle” pattern (characterized by high intakes of alcohol and noodles, and a lack of fruits, whole-grain products, milk, and dairy products) was positively associated with B-Pb. There was no association for a third pattern, “grain and kimchi” (characterized by high intakes of grain and kimchi, and low intakes of fast foods, noodles, bread, and meat) (19). B-Pb concentrations were reported in only 1 of these studies [US study: 6.1 ± 4.2 µg/dL (20)], which is higher than in the present study and might have amplified the relative strength of their results. Together, these studies suggest that adoption of a healthy overall diet in line with WHO recommendations (38) might minimize B-Pb. In the present study, however, there was no association of the “healthy” diet pattern (which is analogous to the “WHO-recommended” and “balanced” patterns in previous studies) with B-Pb in the complete case analysis (and indeed it was predictive of B-Pb ≥5 µg/dL in the imputed dataset). Comparisons of associations of dietary patterns in pregnancy with B-Pb with those from nonpregnant and older adults in previous studies (18–20) may have limited validity: lead absorption, mobilization, and excretion likely differ with physiologic status.

We found that the “confectionery” pattern was associated with lower likelihood of having B-Pb ≥5 µg/dL. Among preschool children in the United States, the consumption of specific foods that would not be untypical of a confectionery-style pattern, such as donuts, and peanut butter and jelly sandwiches, were associated with higher B-Pb (39). The sticky and greasy nature of these food items, however, probably contributed to children's oral lead intakes by additional contamination from their hands having been in contact with residential dust and possibly soil (39, 40). The “confectionery” pattern in the present study is associated with high refined carbohydrate and sugar intake and low intakes of dietary fiber and vitamins and minerals, including calcium and iron (41), and thus does not conform to WHO recommendations (38). Vegetables provide a large proportion of dietary lead in the United Kingdom (42), and therefore a diet lacking in vegetables, as in the “confectionery” pattern, may contribute to lower overall exposure. The association of leafy green vegetables with B-Pb is similar to the observation of an association with a “nutrient-dense” dietary pattern (characterized by dark green and red/orange vegetables) in children in Uruguay (43, 44).

There are limited data available on the lead content of food items and their relative contribution to overall lead intake. Although individual foods such as lead-shot game meat, herbs and spices, and wine each have high concentrations of lead (45–50), it is their relative contribution to the overall lead content of the diet that is of importance. In a general population this contribution is likely to be low, at least for lead-shot game meat and spices, as these are consumed infrequently or in small quantities (51). Cereals and vegetables (specifically leafy vegetables) were identified by the European Food Safety Authority as being most important overall contributors to dietary lead exposure in Europe (7). Similarly, the UK Food Standards Agency identified beverages, bread, and vegetables as contributing the most lead to population exposure from diet (42). Although we did not find any associations of cereal-based food group consumption (“all pasta and rice” and “combined bread and cereals”) with B-Pb, we found a stronger association for “all leafy green and green vegetables,” in accordance with European Food Safety Authority data (7). Lead accumulation from the soil in which plants are grown is thought to be low (52, 53), so that lead contamination from dust and food-processing procedures may be more important. In Korean adolescent girls and adult women there was no association of B-Pb with vegetable intake (54), raising the possibility that local growing and processing conditions may be important. Careful washing of leafy vegetables before consumption could also reduce lead exposure from this source.

It is also possible that other nutrients or components in green leafy vegetables facilitate absorption of lead. Individual nutrients that may be involved in modifying lead absorption or mobilization include iron, vitamin C, zinc, and calcium (11). Iron and calcium intake were previously identified as weakly protective against high B-Pb in pregnant women from ALSPAC (12). However, in the present study we found no consistent associations with the major dietary sources of iron and calcium, and their major sources, meat and milk, respectively, suggesting that translating findings from single-nutrient to food-based recommendations may be inappropriate. The role of calcium status may be important, although calcium supplementation has been shown to have only a small negative effect on B-Pb in pregnant women with adequate dietary calcium intake (13). The effects of iron supplementation in B-Pb in pregnancy in women with compromised iron status is unknown.

This is the first study to report on B-Pb and dietary patterns in pregnancy and further studies are needed to investigate whether any patterns apply specifically to pregnancy, whether associations are region specific, how they relate to the age of the participants, and the relative importance of diet in the overall exposure to lead.

This study has several strengths. First, there are many advantages to the use of dietary patterns and food groups: *1*) it takes into account the synergistic/antagonistic effects of combinations of nutrients and foods; *2*) it can provide information that is more meaningful in translation to public health messages; and *3*) identification of dietary patterns by PCA is less sensitive to inaccuracy and bias in dietary data collection than is assessment of single nutrients. Second, the study provides a valuable addition to the small body of literature on lead and dietary patterns by including a relatively large population of pregnant women, a vulnerable group for whom it is critical to reduce lead exposure to a minimum. Third, in addition to dietary patterns, we were able to look further at specific foods and food groups that might have contributed to increased B-Pb because of being sources of lead or because they provided nutrients that interfered with lead absorption or metabolism.

There are also some study limitations. First, the study was based in a largely urban population in the United Kingdom and may have limited generalizability to other populations of pregnant women both in the United Kingdom and in other countries. Lead exposure was relatively low (55) and this may also limit generalizability to countries with higher exposures. Second, blood was sampled for lead analysis in the first trimester, whereas the dietary data were collected in the third trimester. As the FFQ questions asked about current intakes, causation may be difficult to infer. However, dietary patterns in pregnancy have been shown to be relatively stable (41), and thus we considered these data to be representative of diet throughout pregnancy. Third, misreporting (both under- and overreporting) are inevitable in dietary data collection and this can undermine the validity of associations. Although there are established methods to identify misreporting of energy, those for foods and food groups are much less well understood. Fourth, the subsample of women enrolled in ALSPAC who were included in this study were not completely identical in sociodemographic characteristics to those who were excluded. It is possible that they had higher B-Pb than those who were excluded, and thus inclusion of the whole cohort would have caused the results to tend to towards the null. Fifth, it was not possible to include the effect of meal patterns and eating occasions [relative lead absorption is greater in the fed state than when fasted (56)]. Sixth, residual confounding could be important in the few associations that were observed, which could have occurred by chance, as well as in null results.

In conclusion, we found evidence for a positive association between the consumption of green leafy vegetables and B-Pb in pregnant women. Other nutrients in green leafy vegetables are likely to be beneficial, so rather than avoidance, thorough washing of these items to remove any contamination before consumption is advisable. Hand-washing before eating to reduce cross-contamination is also sound advice. Further investigation in other cohorts in other locations is essential. We found limited evidence of an association between women's typical diet and B-Pb during pregnancy, and there was some indication of instability between findings from complete cases compared with multiple imputation and a categoric outcome compared with a continuous outcome. Therefore, our findings are unlikely to be a major consideration in revision of existing dietary guidance for pregnant women, who are advised to adopt a healthy diet in pregnancy, with a variety of foods consumed in moderation. Exposures from other sources, including water, should also remain a focus of attention for public health measures.

## Supplementary Material

nxz023_Supplemental_File

## Notes

The UK Medical Research Council and the Wellcome Trust (grant 102215/2/13/2) and the University of Bristol currently provide core support for ALSPAC. In addition, this study was funded by a seed grant from the Biomedical Research Unit (BRU), University of Bristol (PI: KK). CMT was supported by a Wellcome Trust Career Re-entry Fellowship (grant 104077/Z/14/Z).

Author disclosures: CMT has received funding from Nestlé Nutrition for work unconnected with this study. The other authors have no conflicts of interest to declare.

Supplemental Tables 1–6 and Supplemental Methods are available from the “Supplementary data” link in the online posting of the article and from the same link in the online table of contents at https://academic.oup.com/jn/.

Abbreviations used: ALSPAC, Avon Longitudinal Study of Parents and Children; B-Pb, blood Pb concentration; PCA, principal components analysis.
