# Supplementary material for: Dietary Patterns Are Not Consistently Associated with Variability in Blood Lead Concentrations in Pregnant British Women
Source: J Nutr. 2019 Apr 10;149(6):1027–36. doi: 10.1093/jn/nxz023 (PMC6543203; doi:10.1093/jn/nxz023)
Supplement: nxz023_Supplemental_File [file nxz023_supplemental_file.pdf]

Supplemental material

Supplemental Methods

Collection, storage and analysis of blood samples

Whole blood samples were collected in acid-washed vacutainers (Becton and Dickinson, Oxford, UK) and stored in the original tube at 4°C at the collection site before being transferred to the central Bristol laboratory within 1–4 days. Samples were at ambient temperature during transfer (up to 3 h). They were then stored at 4°C until analysis. Details of the analysis have been reported (1, 2). Inductively-coupled plasma mass spectrometry in standard mode (R. Jones, Centers for Disease Control and Prevention (CDC), Bethesda, MD, USA; CDC Method 3009.1) was used to measure blood concentrations with appropriate quality controls. One sample had a lead level below the limit of detection (0.24 µg/dL). This sample was assigned a value of 0.7 times the lower limit of detection (limit of detection/√2) to reflect the log-normal distribution (3, 4).

Potential confounders

Potential confounders included measures of socio-economic positioning (SEP), body mass index (BMI), estimated energy intake, lifestyle indicators (alcohol consumption and smoking status during pregnancy) and hemoglobin levels. Indicators of SEP were: age at pregnancy (categorised as ≤19, 20–24, 25–29, 30–34, ≥35 years), highest level of educational attainment (none/Certificate of School Education, vocational/Ordinary level, Advanced level and above), and Townsend score, a measure of material deprivation based on geographical area, incorporating census-based data on unemployment, non-car ownership, non-home ownership and household overcrowding (5) (quartiles from least deprived to most deprived). Data on BMI (from height and pregnancy weight; kg/m^2), smoking status (yes/no) and alcohol

consumption (yes/no) during the first trimester were obtained from questionnaires completed during pregnancy. Energy intake (kJ/day) was estimated from the FFQ at 32 weeks' gestation (6). Hemoglobin levels (g/dL) were extracted from obstetric clinic records. The first recorded level was used to correspond with the gestational time of the blood sample for lead analysis.

Supplemental references

1. Golding J, Steer CD, Hibbeln JR, Emmett PM, Lowery T, Jones R. Dietary predictors of maternal prenatal blood mercury levels in the ALSPAC birth cohort study. Environmental Health Perspectives 2013;121(10):1214-8. doi: 10.1289/ehp.1206115.
2. Taylor CM, Kordas K, Golding J, Emond AM. Data relating to prenatal lead exposure and child IQ at 4 and 8 years old in the Avon Longitudinal Study of Parents and Children. Neurotoxicology 2017;62:224-30. doi: 10.1016/j.neuro.2017.07.025.
3. Hornung R, Reed LD. Estimation of average concentration in the prescence of nondetectable values. Applied Occupational and Environmental Hygiene 1990;5:46-51.
4. Centers for Disease Control and Prevention. Third National Report on human exposure to environmental chemicals, 2005.
5. Townsend P, Phillimore P, Beattie A. Health and deprivation: inequality and the north. London: Croom Helm, 1988.
6. Brion MJ, Ness AR, Rogers I, Emmett P, Cribb V, Davey Smith G, Lawlor DA. Maternal macronutrient and energy intakes in pregnancy and offspring intake at 10 y: exploring parental comparisons and prenatal effects. American Journal of Clinical Nutrition 2010;91(3):748-56. doi: 10.3945/ajcn.2009.28623.

Supplemental Table 1 Food groups and individual foods as combined for the presented analyses

Food group	Individual foods in group
Meats	Poultry, red meat, offal,
Fish	White fish, oily fish. shell fish
Pulses	Pulses, bakes beans
Nuts	Nuts, tahini
Soybean products	Bean curd, soya/non-meat
Root vegetables	Carrots, boiled/baked/roasted potatoes, other root vegetables
Leafy greens and green vegetables	Peas, leafy greens, other green vegetables, salad
Breads and cereals	Oat cereal, bran cereal, other cereal, crispbread
Cakes and biscuits	Cakes, buns, biscuits
Pasta and rice	Pasta, rice
Pies/pastries	Pizza, pies, pastries

Supplemental Table 2 Blood lead concentrations of ALSPAC participants by indicators of socioeconomic positions and lifestyle: imputed data

Variable	Observations included in sample (%)	% B-Pb <5 μg/dL	% B-Pb ≥5 μg/dL	OR (95% CI)	*P* value
Maternal age at pregnancy (years)					<0.001
≤19	5.2	4.9	4.1	1.00	
20 to <25	19.8	19.9	16.9	1.02 (0.73, 1.42)	
25 to <30	38.2	39.0	37.0	1.15 (0.81, 1.60)	
30 to <35	26.6	26.8	29.2	1.31 (0.90, 1.90)	
≥35	10.2	9.3	12.7	1.64 (1.06, 2.54)	
Maternal education (at pregnancy)					<0.001
None/CSE	36.3	32.0	26.1	1.00	
Vocational/O-level	53.6	56.6	56.5	1.22 (1.04, 1.43)	
A-level and above	10.2	11.4	17.4	1.86 (1.54, 2.24)	
Townsend score (at pregnancy)					<0.001
1	30.3	32.7	25.6	1.00	
2	18.2	18.6	17.3	1.19 (1.00, 1.43)	
3	26.9	25.9	30.1	1.48 (1.21, 1.80)	
4	24.6	22.9	27.1	1.51 (1.19, 1.93)	
Smoking status (1st trimester)					<0.001
No	70.3	76.2	66.8	1.00	
Yes	29.7	23.8	33.2	1.60 (1.33, 1.91)	
Alcohol consumption (1st trimester)					<0.001
No	47.4	46.5	41.4	1.00	
Yes	52.6	53.5	58.6	1.23 (1.08, 1.41)	
BMI (pre-pregnancy)					0.431
Normal/underweight	78.0	79.0	79.8	1.00	
Overweight	15.8	15.2	15.2	0.99 (0.82, 1.19)	
Obese	6.2	5.8	5.0	0.85 (0.61, 1.21)	
Vegetarian diet					<0.001
Never	85.9	86.6	83.3	1.00	
In the past	9.0	8.4	10.0	1.23 (0.99, 1.54)	
Present	5.1	5.0	6.7	1.41 (1.09, 1.82)	

Educational attainment; none/Certificate of School Education, vocational/Ordinary level, Advanced level and above.

Townsend score is a measure of maternal deprivation: 1 is the least deprived, score 4 the most deprived (5).

BMI (body mass index): normal/underweight <24.9; overweight 25.0–29.9; obese >30.0 kg/m^2.

Supplemental Table 3 Categorical blood lead levels for cases with blood lead concentration measurements by dietary patterns: imputed data

Pattern	Quartile	%		OR (95% CI)		
		B-Pb <5 µg/dL	B-Pb ≥5 µg/dL	Unadjusted Model 1	Adjusted Model 2	Adjusted Model 3
Healthy	1	26.6	22.4	1.00		
	2	25.3	23.6	1.11 (0.95, 1.30)	1.19 (1.02, 1.40)	1.20 (1.01, 1.42)
	3	24.5	25.3	1.23 (0.84, 1.29)	1.35 (1.11, 1.63)	1.33 (1.08, 1.66)
	4	23.5	28.7	1.45 (0.82, 1.43)	1.58 (1.27, 1.97)	1.52 (1.17, 1.97)
				P-trend<0.001	*P*-trend<0.001	*P*-trend<0.001
Traditional	1	25.5	24.5	1.00		
	2	24.9	25.1	1.05 (0.87, 1.26)	1.06 (0.88, 1.28)	1.07 (0.89, 1.29)
	3	24.9	24.8	1.04 (0.74, 1.29)	1.06 (0.85, 1.34)	1.08 (0.86, 1.35)
	4	24.8	25.8	1.08 (0.82, 1.43)	1.12 (0.84, 1.48)	1.13 (0.85, 1.50)
				P-trend=0.621	*P*-trend=0.506	*P*-trend=0.452
Processed	1	24.0	26.7	1.00		
	2	24.6	24.7	0.91 (0.76, 1.08)	0.95 (0.79, 1.15)	0.95 (0.79, 1.15)
	3	25.3	24.0	0.85 (0.69, 1.05)	0.93 (0.74, 1.16)	0.94 (0.74, 1.19)
	4	26.1	24.6	0.85 (0.69, 1.04)	0.94 (0.74, 1.20)	0.94 (0.72, 1.22)
				P-trend=0.112	*P*-trend=0.604	*P*-trend=0.609
Confectionery	1	23.9	29.7	1.00		
	2	245	25.4	0.83 (0.71, 0.98)	0.86 (0.73, 1.02)	0.88 (0.75, 1.05)
	3	25.4	22.8	0.72 (0.60, 0.87)	0.76 (0.61, 0.94)	0.79 (0.63, 0.99)
	4	26.3	22.1	0.68 (0.54, 0.85)	0.72 (0.55, 0.95)	0.76 (0.57, 1.03)
				P-trend<0.001	*P*-trend=0.016	*P*-trend=0.061
Vegetarian	1	25.0	23.70	1.00		
	2	25.2	23.4	0.98 (0.83, 1.15)	0.96 (0.82, 1.14)	1.01 (0.86, 1.19)
	3	25.1	24.9	1.04 (0.84, 1.29)	1.01 (0.81, 1.25)	1.09 (0.88, 1.36)
	4	24.7	28.1	1.20 (0.96, 1.50)	1.10 (0.88, 1.38)	1.15 (0.91, 1.45)
				P-trend=0.117	*P*-trend=0.592	*P*-trend=0.226

Model 2 adjusted for maternal age, maternal education, Townsend score + BMI and energy intake + alcohol consumption and smoking status during first trimester + hemoglobin levels.
Model 3 adjusted for maternal age, maternal education, Townsend score + BMI and energy intake + alcohol consumption and smoking status during first trimester + hemoglobin levels + dietary pattern scores.

Supplemental Table 4 Categorical blood lead concentrations of cases with valid blood lead level measurements by food group: imputed data

	%		OR (95% CI)	
	B-Pb <5 µg/dL	B-Pb ≥5 µg/dL	Unadjusted model 1	Adjusted model 2
**All meats combined**				
≤ Once in 2 weeks	14.1	15.9	1.00	
≤3 times per week	63.5	61.9	0.86 (0.72, 1.04)	0.92 (0.76, 1.12)
≥4 times per week for at least one group	22.4	22.2	0.88 (0.66, 1.15)	0.94 (0.71, 1.25)
			P-trend=0.423	*P*-trend=0.722
**All fish**				
≤ Once in 2 weeks	51.6	50.5	1.00	
≥1 to 3 times per week	44.7	45.0	1.03 (0.87, 1.22)	1.04 (0.86, 1.25)
≥4 to 7 times per week	3.7	4.6	1.26 (0.92, 1.73)	1.27 (0.89, 1.82)
			P-trend=0.419	*P*-trend=0.406
**Milk (glasses per day)**				
None/rarely	42.0	46.8	1.00	
1 to 2 glasses per day	47.8	44.9	0.84 (0.74, 0.96)	0.87 (0.76, 1.00)
≥3 glasses per day	10.2	8.3	0.73 (0.58, 0.91)	0.77 (0.61, 0.98)
			P-trend=0.003	*P*-trend=0.019
**Calcium intake (quartiles)**				
1	24.4	27.4	1.00	
2	24.9	24.8	0.89 (0.76, 1.03)	0.87 (0.73, 1.05)
3	25.0	23.9	0.85 (0.69, 1.05)	0.83 (0.63, 1.09)
4	25.7	23.9	0.83 (0.66, 1.04)	0.81 (0.57, 1.15)
			P-trend=0.125	*P*-trend=0.244
**All pulses combined**				
≤ Once in 2 weeks	12.3	13.3	1.00	
≤3 times per week	79.8	77.8	0.91 (0.76, 1.08)	0.94 (0.79, 1.13)
≥4 times per week for at least one group	8.0	8.9	1.04 (0.87, 1.59)	1.07 (0.85, 1.45)
			P-trend=0.992	*P*-trend=0.795
**All nuts combined**				
Never/rarely	69.6	67.1	1.00	
≤ Once in 2 weeks	21.6	22.9	1.10 (0.95, 1.27)	0.94 (0.79, 1.13)
≥1 to 3 times per week	8.8	10.0	1.17 (0.87, 1.59)	1.07 (0.80, 1.45)
			P-trend=0.208	P-trend=0.616
**Soybean products**				
Never or rarely	92.0	89.4	1.00	
≤ Once in 2 weeks	8.0	10.6	1.36 (1.11, 1.67)	1.23 (0.99, 1.52)
			P-trend=0.003	*P*-trend=0.063
**Root vegetables**				
Never or rarely	5.9	5.7	1.00	
≤ One to 3 times per week per food	59.3	58.2	1.02 (0.74, 1.39)	1.05 (0.77, 1.44)

	%		OR (95% CI)	
	B-Pb <5 µg/dL	B-Pb ≥5 µg/dL	Unadjusted model 1	Adjusted model 2
≥4 to 7 times per week	34.8	36.1	1.08 (0.71, 1.65)	1.18 (0.77, 1.81)
			P-trend=0.615	*P*-trend=0.309
**All leafy green and green vegetables**				
≤1 to 3 times per week	25.2	22.4	1.00	
≥4 times per week	74.9	77.6	1.16 (0.94, 1.43)	1.16 (0.94, 1.44)
			P-trend=0.160	*P*-trend=0.158
**Combined breads and cereals**				
≤ Once a week	12.4	14.3	1.00	
≤One to 3 times per week per food	28.5	29.2	0.90 (0.70, 1.41)	0.94 (0.73, 1.21)
≥4 to 7 times per week	59.1	56.6	0.84 (0.65, 1.11)	0.91 (0.66, 1.25)
			P-trend=0.208	*P*-trend=0.588
**All cakes and biscuits**				
≤ Once a week	17.2	21.6	1.00	
≤ One to 3 times per week per food	49.0	49.0	0.80 (0.67, 0.95)	0.83 (0.69, 0.99)
≥4 to 7 times per week	33.7	29.4	0.69 (0.55, 0.88)	0.75 (0.57, 0.97)
			P-trend=0.004	*P*-trend=0.588
**All pies and pastries**				
Never or rarely	20.3	22.7	1.00	
≤ Once in 2 weeks	52.5	52.2	0.89 (0.76, 1.03)	0.91 (0.79, 1.07)
≥1 to 3 times per week	27.2	25.0	0.82 (0.67, 1.01)	0.89 (0.71, 1.11)
			P-trend=0.062	*P*-trend=0.301
**All pasta and rice**				
Never or rarely	14.4	11.1	1.00	
≤ Once in 2 weeks	31.9	28.1	1.16 (0.94, 1.44)	1.16 (0.94, 1.43)
≥1 to 3 times per week	53.7	61.0	1.50 (1.19, 1.87)	1.45 (1.14, 1.84)
			P-trend<0.001	*P*-trend=0.001

Model 2 adjusted for maternal age, maternal education, Townsend score + BMI and energy intake + alcohol consumption and smoking status during first trimester + hemoglobin levels.

Supplemental Table 5 Continuous log blood lead levels for cases with valid blood lead concentration measurements by dietary patterns (complete case analysis, *n*=2,167)

Pattern	Beta coefficient (95% CI)	
	Unadjusted model	Adjusted model
Healthy	0.002 (-0.014, 0.018), p=0.815	0.013 (-0.006, 0.032), p=0.176
Traditional	0.004 (-0.012, 0.020), p=0.328	0.010 (-0.005, 0.026), p=0.197
Processed	-0.008 (-0.025, 0.008), p=0.324	0.004 (-0.015, 0.023), p=0.662
Confectionery	-0.030 (-0.467, -0.014), p<0.001	-0.011 (-0.031, 0.007), p=0.210
Vegetarian	0.030 (0.014, 0.045), p<0.001	0.011 (-0.004, 0.025), p=0.142

Model adjusted for maternal age, maternal education, Townsend score + BMI and energy intake + alcohol consumption and smoking status during first trimester + hemoglobin levels.

Beta coefficients are log-transformed.

Supplemental Table 6 Continuous log blood lead concentrations of cases with valid blood lead level measurements by food group (complete case analysis, *n*=2,167)

	Beta coefficient (95% CI)	
	**Unadjusted model**	**Adjusted model**
**All meats combined**		
≤ Once in 2 weeks	-	-
≤3 times per week	-0.061 (-0.107, -0.014), p=0.011	-0.023 (-0.068, 0.020), p=0.292
≥4 times per week for at least one group	-0.044 (-0.097, 0.009), p=0.105	0.020 (-0.032, 0.071), p=0.463
**All fish**		
≤ Once in 2 weeks	-	-
≥1 to 3 times per week	0.021 (-0.069, -0.006), p=0.021	-0.024 (-0.054, 0.007), p=0.129
≥4 to 7 times per week	0.103 (0.019, 0.186), p=0.016	0.104 (0.025, 0.182), p=0.010
**Milk (glasses per day)**		
None/rarely	-	-
1 to 2 glasses per day	-0.048 (-0.082, -0.015), p=0.004	-0.037 (-0.068, -0.005), p=0.021
≥3 glasses per day	-0.077 (-0.137, -0.018), p=0.011	-0.059 (-0.115, -0.002), p=0.042
**Calcium intake (quartiles)**		
1	-	-
2	-0.030 (-0.074, 0.015), p=0.189	-0.011 (-0.054, 0.032), p=0.626
3	-0.027 (-0.071, 0.018), p=0.245	-0.008 (-0.057, 0.039), p=0.724
4	-0.070 (-0.115, -0.025), p= 0.002	-0.015 (-0.074, 0.044), p=0.612
**All pulses combined**		
≤ Once in 2 weeks	-	-
≤3 times per week	-0.037 (-0.085, 0.010), p=0.124	-0.030 (-0.075, 0.015), p=0.189
≥4 times per week for at least one group	-0.021 (-0.091, 0.048), p=0.544	-0.017 (-0.083, 0.049), p=0.616
**All nuts combined**		
Never/rarely	-	-
≤ Once in 2 weeks	-0.037 (-0.085, 0.010), p=0.124	-0.030 (-0.075, 0.015), p=0.189
≥1 to 3 times per week	-0.021 (-0.091, 0.048), p=0.544	-0.017 (-0.083, 0.049), p=0.616
**Soybean products**		-
Never or rarely	-	-
≤ Once in 2 weeks	0.045 (-0.009, 0.098), p=0.104	0.021 (-0.030, 0.072), p=0.416
**Root vegetables**		
Never or rarely	-	-
≤ One to 3 times per week per food	-0.017 (-0.097, 0.061), p=0.668	-0.010 (-0.084, 0.065), p=0.797
≥4 to 7 times per week	-0.060 (-0.141, 0.021), p=0.148	-0.020 (-0.098, 0.058), p=0.618
**All leafy green and green vegetables**		
≤1 to 3 times per week	-	-
≥4 times per week	-0.017 (-0.120, 0.087), p=0.754	0.007 (-0.094, 0.107), p=0.896
**Combined breads and cereals**		
≤ Once a week	-	-
≤One to 3 times per week per food	-0.082 (-0.136, -0.028), p=0.003	-0.050 (-0101, 0.001), p=0.053
≥4 to 7 times per week	-0.098 (-0.148, -0.049), p<0.001	-0.043 (-0.091, 0.005), p=0.081
**All cakes and biscuits**		
≤ Once a week	-	-
≤ One to 3 times per week per food	-0.089 (-0.132, -0.046), p<0.001	-0.065 (-0.107, -0.024), p=0.002
≥4 to 7 times per week	-0.126 (-0.172, -0.080), p<0.001	-0.073 (-0.121, -0.026), p=0.002
**All pies and pastries**		
Never or rarely	-	-
≤ Once in 2 weeks	-0.019 (-0.059, 0.022), p=0.370	-0.004 (-0.042, 0.034), p=0.840
≥1 to 3 times per week	-0.061 (-0.108, -0.013), p=0.012	-0.036 (-0.083, 0.010), p=0.128
**All pasta and rice**		
Never or rarely	-	-
≤ Once in 2 weeks	-0.016 (-0.07, 0.042), p=0.591	-0.003 (-0.057, 0.052), p=0.917
≥1 to 3 times per week	0.021 (-0.033, 0.074), p=0.449	0.034 (-0.019, 0.087), p=0.210

Model adjusted for maternal age, maternal education, Townsend score + BMI and energy intake + alcohol consumption and smoking status during first trimester + hemoglobin levels.

Beta coefficients are log-transformed.
